# Reduced apoptotic levels in squamous but not basal cell carcinomas correlates with detection of cutaneous human papillomavirus

**DOI:** 10.1038/sj.bjc.6600431

**Published:** 2002-08-01

**Authors:** S Jackson, L Ghali, C Harwood, A Storey

**Affiliations:** Cancer Research UK, Skin Tumour Laboratory, Centre for Cutaneous Research, 2 Newark Street, London E1 2AT, UK

**Keywords:** HPV, squamous, UVB, apoptosis, Bak

## Abstract

We have investigated the apoptotic levels and expression of the apoptotic inducer Bak in non-melanoma skin cancers. Squamous cell carcinomas of known human papillomavirus status from immunocompetent patients were analysed for the expression of the Bak protein, and the expression profile was compared both to the presence of apoptotic cells and the proliferation marker Ki-67. We demonstrate an inverse correlation between human papillomavirus positivity and Bak expression in squamous cell carcinomas, with concomitantly fewer apoptoic cells being detected in the human papillomavirus positive tumours. Bak expression was not observed in basal cell carcinomas irrespective of human papillomavirus status, suggesting that Bak only plays a role in signalling apoptosis in squamous, but not basal, cell cancers. No differences were observed in the proliferation rates between papillomavirus positive and negative squamous cell tumours. However, a significant decrease in the number of apoptotic cells was observed in human papillomavirus-positive squamous cell carcinomas which suggests that the virus may have significantly altered the relationship between proliferation and apoptosis in a proportion of these tumours.

*British Journal of Cancer* (2002) **87**, 319–323. doi:10.1038/sj.bjc.6600431 www.bjcancer.com

© 2002 Cancer Research UK

## 

Non-melanoma skin cancer (NMSC) is the commonest malignancy in Caucasian populations with around 40 000 new cases arising each year in the UK and about 1 million in the USA. The cumulative effect of solar ultra-violet B (UVB) radiation at 280–320 nm wavelengths is a key environmental risk factor in the aetiology of the disease (de Gruijl and van der Leun, 1994; Kraemer, 1997; Miralles *et al*, 1998). In addition to UVB, other factors such as immunological status, genetic predisposition and infection by human papillomavirus (HPV) may also contribute towards NMSC development (Proby *et al*, 1996). These cancers typically arise at body sites exposed to UVB and recent epidemiological studies have revealed that about 30% of squamous cell carcinomas (SCC) in immunocompetent individuals harbour HPV DNA (Harwood *et al*, 2000, and references therein). In iatrogenically immunosuppressed organ transplant recipients the carriage of HPV DNA in SCCs rises dramatically to about 80% with a wide spectrum of HPV types being present (Harwood *et al*, 2000). Whether the virus plays an active role in the development of lesions carrying HPV DNA is currently the focus of many investigations.

Acute UVB exposure leads to the formation of ‘sunburn’ cells within the epidermis which display characteristics, such as condensed nuclei, that is typical of apoptotic cells (Young, 1987; Schwarz *et al*, 1995). The apoptotic response of cells within the epidermis is in part dependent on the p53 tumour suppressor protein (Brash *et al*, 1996; Ziegler *et al*, 1996), however other p53-independent mechanisms have also been identified (Gniadecki *et al*, 1997). Whilst the role of p53 in protecting the epidermis from UV-induced skin carcinomas is clearly established in murine models (Donehower *et al*, 1992; Ananthaswamy *et al*, 1997; Jiang *et al*, 1999), there is conflicting evidence as to the protective role of p53 in adult human skin. In particular, the suggested lack of pre-cancerous potential of the many clonal patches of p53 mutant cell clones within the epidermis (Jonason *et al*, 1996; Ren *et al*, 1996a,b), together with the lack of increase in NMSC frequency in Li-Fraumeni patients (Malkin *et al*, 1990), suggests that both p53-dependent and independent surveillance mechanisms may be critical in eliminating UVB damaged cells. P53 immunostaining, which is often indicative of a dysfunction in the p53 pathway, is observed in about 40% of SCCs (Mcgregor *et al*, 1997). However, this aberrant p53 expression does not correlate with the number of apoptotic cells observed in SCCs (Wikonkal *et al*, 1997; Jackson *et al*, 2000). The persistence of cells which have acquired somatic mutations as a direct result of UV-induced photoproduct formation (Herzinger *et al*, 1995) may lead to the propagation of deleterious mutations and ultimately to carcinogenesis. In addition to the epidemiological studies on the presence of HPV DNA in these tumours, we have been studying the molecular activities of the viral proteins to determine whether they possess functions that are consistent with a role in tumour formation. We have recently demonstrated that the E6 protein from a diverse spectrum of HPV types is able to efficiently inhibit apoptosis in cell culture systems in response to UVB damage (Jackson and Storey, 2000). In addition, we have shown that the Bak protein, an apoptogenic member of the Bcl-2 family, is induced to high levels in the epidermis in response to UVB that in turn leads to apoptosis. The E6 protein of cutaneous HPVs efficiently targets Bak for proteolytic degradation, both in monolayer cell cultures and in complex models of regenerated epithelium (Jackson *et al*, 2000). This suppression of apoptosis suggests a mechanism by which the virus may play a general role in promoting SCC development. To investigate this further, we have analysed a series of both HPV positive and negative SCCs and basal cell carcinomas (BCCs) for Bak expression and correlated this with the proliferative potential of the tumour and apoptotic levels.

## MATERIALS AND METHODS

### Patients and tumours

Lesions comprised of 24 CIS/SCCs from 20 patients and 16 BCC lesions derived from 16 patients. All patients were immunocompetent and tumour diagnosis was confirmed histologically. All lesions were 1–1.5 cm in size. Each sample was bisected and half frozen at −70°C at the time of surgery. Ethical approval for this study was obtained from the East London and City Health Authority Research Ethics Committee.

### HPV typing of lesions

DNA from frozen tumour tissue were subjected to HPV typing using a degenerate PCR strategy that we have previously demonstrated to detect all known HPV types (Harwood *et al*, 2000).

### Western blotting

Proteins were extracted from 5 μm section by resuspending the tissue in 100 μl 2×SDS sample buffer, vortexing and then heating to 100°C for 5 min. Ten μl of each sample was then electrophoresed on 12% SDS polyacrylamide gels and either stained with coomassie blue or transferred to PVDF membrane for Western blot analysis. Primary antibodies were diluted according to manufacturers instructions. The secondary antibody was a HRP-coupled rabbit anti-mouse serum used at 1 : 1000 dilution (Dako) and detection of proteins was by ECL-Plus (Amersham Pharmacia Biotech).

### Antibodies and immunohistochemistry

Antibodies for Western blotting were: Bak (Ab-2, Calbiochem), W6/32 (MHC class 1, Serotech), actin (Dako).

HPV positive and HPV-negative SCCs tumours and BCCs were investigated using standard immunohistochemical techniques for protein expression of Bak, Ki-67 and by apoptosis as judged by TUNEL staining. Four μm thick formalin fixed paraffin embedded tissue sections were deparaffinized and rehydrated through graded alcohols to water. A minimum of two sections were examined for each parameter. Endogenous peroxidase activity was blocked by incubation with 0.03% H_2_O_2_ for 10 min. The sections which were used to detect Ki-67 (Dako Ltd, 1 : 800 dilution) expression were returned to water and then immersed in preheated 10 mM citrate buffer, pH 6.0 and microwaved for 8 min, then blocked with horse serum and incubated in the primary antibody overnight at 4°C. For detection of Bak protein expression (Ab-2, Calbiochem, 1 : 100 dilution), the slides were immersed in 10 mM citrate buffer and then boiled in a microwave for a total of 15 min consisting of three 5 min periods. The slides were then left to cool for 20 min before taking them back to water and then blocking with horse serum. The primary antibody was then applied for 4 h at room temperature. For both antibodies the rest of the procedure was the same. Briefly, the sections were rinsed with Tris buffer saline (TBS) and the biotinylated antibody from the universal streptavidin-biotin kit (Vector Lab) applied for 30 min, before rinsing with TBS. The third layer peroxidase labelled streptavidin was applied for another 30 min and the sections rinsed again before developing using diaminbenzidine (DAB). The sections were then lightly counterstained using Gill's haematoxylin, dehydrated in graded alcohols, cleared in xylene and mounted using DePex (Metk Ltd). TUNEL staining of tissue was carried out according to manufacturers instructions (Promega, DeadEnd colorimetric apoptosis detection system), using a 5 μg ml^−1^ Proteinase K incubation for 5 min.

Each tissue section was then scored by two independent assessors without knowledge of the clinical details or HPV status of the lesion.

The entire tumour section was assessed and analysis was carried out on a quantitative basis with the following grading applying throughout: −: no staining; positive staining was scored follows: + : </= 5% of tumour cells positive; ++ : >5–25%; +++ : 25–50%; ++++ : >50–75%; +++++ : >75–100%. Statistical analysis was performed using Fishers exact test.

## RESULTS

HPV positive and negative tumours were investigated using standard immunohistochemical techniques for expression of the Bak protein, the proliferation marker Ki-67 and apoptosis as judged by TUNEL staining. Three out of 10 of the HPV positive biopsies were carcinoma *in situ* (CIS) and the remaining seven were SCCs; the HPV-negative tumours comprised two CIS and 12 SCC samples. Of the HPV-positive samples, five displayed Bak staining and this was restricted to the lowest scoring index with only a few cells being scored positive in the entire tumour section, whilst more of the HPV negative SCCs showed staining which was often of a higher scoring index, but did not reach statistical significance in our study (*P*=0.440). The Bak staining was cytoplasmic in all cases which is consistent with its subcellular mitochondrial localisation. Concomitant with Bak expression, only one sample showed evidence of apoptosis, but staining with Ki-67, a large protein present in the nuclei of all proliferating cells, revealed however that the tumours were highly proliferative (Table 1

**Table 1:**
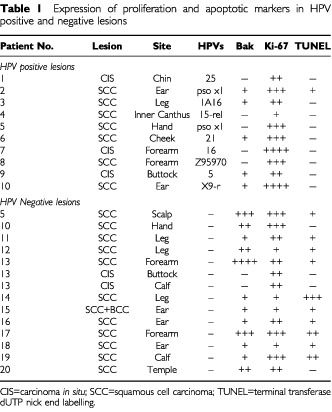
Expression of proliferation and apoptotic markers in HPV positive and negative lesions

). In marked contrast, 11 out of 14 of the HPV-negative tumours displayed Bak staining, with three samples having strong staining throughout the tumour (Table 1). A most notable finding was that there was an increase in TUNEL positivity that accompanied the increased Bak staining in the HPV-negative SCCs compared to the HPV-positive samples (*P*=0.008), whilst no differences were observed in Ki-67 staining index relative to the HPV status. Bak staining was restricted to the tumour material and was never detected in the overlying epidermis or stroma.

**Figure 1:**
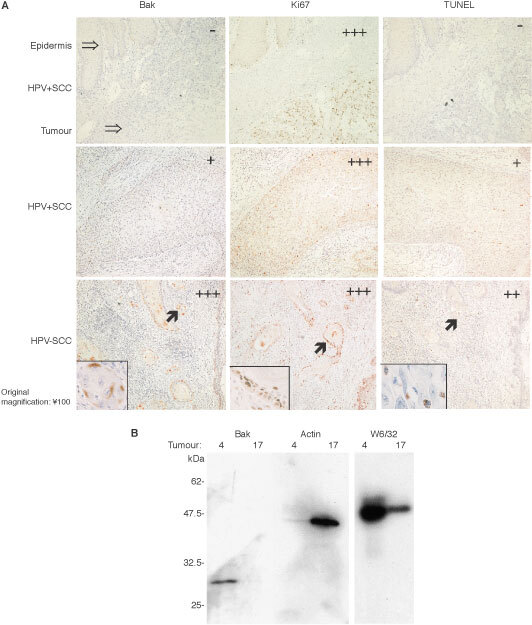
(**A**) Immunohistochemical detection of Bak, Ki-67 and TUNEL in SCCs. Shown are serial tumour sections that were analysed for the expression of the pro-apoptotic protein Bak, the proliferation marker Ki-67 and the proportion of cells undergoing apoptosis, as judged by TUNEL. The scoring system employed is as described in the text. Original magnification 100×. (**B**) Western blot analysis of Bak positive and negative SCCs. Proteins extracted from equal amounts of tumour material were subjected to SDS–PAGE and Western blotting for Bak, actin and MHC class 1 (W6/32) expression. Tumours 4 and 17 were selected as being representative of Bak negative and positive immunohistochemical staining.

Representative examples of different grades of Bak, Ki-67 and TUNEL staining are shown in Figure 1A

. In order to verify that the increased Bak immunohistochemical staining was reflective of high Bak protein levels in the tumour, two 5 μm sections of similar sized tumour were cut from Bak positive and negative lesions, patients 17 and 4 respectively. The extracted proteins were electrophoresed on SDS–PAGE gels and the proteins transferred to PVDF membranes for Western blot analysis. Coomassie blue staining of the samples indicated that they contained equal amounts of protein, but in addition to Bak the membranes were also re-probed for actin and MHC class 1. Western blotting confirmed that Bak was easily detected in the section that showed positive immunohistochemical Bak staining (from lesion 17), but not in the Bak negative tumour sections (from lesion 4) (Figure 1B). Whilst both tumours showed MCH class 1 staining, we note that the Bak negative lesion expressed actin whilst in the Bak positive lesion actin expression was much lower, suggesting a degree of heterogeneity between the tumour samples. There was no clear correlation between Bak expression and TUNEL positivity (Table 1). This may be due to the expression of apoptotic inducers such as Bak being a prerequisite to apoptotic cell formation and also that host cell protein synthesis is frequently shut down as cells as they undergo apoptosis.

We next investigated a series of BCCs and compared our finding to those of the SCC samples. Bak was not detected in any of the seven HPV-positive BCCs compared to five out of 10 SCCs (*P*=0.003), or nine HPV-negative BCC samples, in comparison to 11 out of 14 HPV positive samples (*P*=0.0005). All the HPV-positive and negative BCCs showed evidence of proliferation, but no marked differences in Ki-67 staining were observed between the two groups. In addition, only one out of seven HPV positive BCC samples were TUNEL positive. A significant difference was observed however between the TUNEL staining of the HPV negative BCCs and SCCs, where only two out of nine BCCs were TUNEL positive compared to 10 out of 14 of the SCCs (*P*=0.0266) (Table 2

**Table 2:**
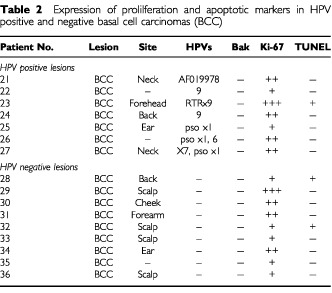
Expression of prolilferation and apoptotic markers in HPV positive and negative basal cell carcinomas (BCC)

).

## DISCUSSION

Our recent findings that a diverse spectrum of HPV types were able to inhibit apoptosis (Jackson and Storey, 2000), coupled with the fact that UVB is a potent inducer of the Bak protein and that the HPV E6 protein abrogates Bak function (Jackson *et al*, 2000), suggested that the virus may be altering the balance between apoptosis and proliferation in HPV-containing lesions. To test this hypothesis, we have investigated a series of HPV positive and negative SCCs for apoptosis and proliferation. We demonstrate that the HPV positive lesions displayed little or no Bak staining and had few apoptotic cells whilst still maintaining a high proliferative potential. In distinct contrast, the HPV negative tumours were more heterogeneous and frequently showed higher levels of Bak expression and apoptosis. We conclude from these studies that HPVs may significantly alter the profile of apoptotic inducers such as Bak and that the rate of apoptosis has been altered in the HPV-positive tumours. Such a perturbation may have significant clinical implications for the development of such lesions since both HPV positive and negative SCCs were highly proliferative as assessed by Ki-67 immunoreactivity (Arends *et al*, 1994). Whether such an effect influences the accumulation of deleterious mutations induced by UVB (Griffiths *et al*, 1998) or alters the pattern of chromosome loss (Quinn *et al*, 1994) in these tumours remains to be determined. We have also noted that HPV positive and negative tumours can occur in the same individuals, such as patients 5 and 10 in Table 1. Whilst this may indicate that the HPV may be a passenger in such lesions, an alternative explanation and one that is common to other virally associated tumours, is that the tumour may develop in the absence of viral infection through an alternative mechanism that involves the accumulation of mutations in critical cellular genes.

Although HPV DNA copy number in such lesions is high enough to be detected by Southern blotting (see Proby *et al*, 1996 for an overview), we have employed a degenerate PCR-based approach that is capable of detecting all known HPV types that does not require large amounts of DNA and a prior knowledge of the HPV DNA status. This methodology is not as sensitive as PCR using type-specific primers and does not enable us to estimate accurately the copy number of the virus in the lesions and whether all cells contain virus and express viral proteins. Future experiments aimed at determining the level and pattern of E6 gene expression in these lesions will be most informative.

Whether a higher viral load coupled with increased E6 expression would further alter Bak expression or apoptotic rates remains to be determined. We also noted that the HPV samples that were positive for Bak expression by immunohistochemistry displayed only a few cells that scored positive. Whether this reflects a heterogeneous expression of the HPV genes within the tumour remains to be investigated in greater detail.

As none of the BCC tumours in our study expressed Bak our findings also suggest that this mechanism of apoptosis in NMSC is restricted to SCCs. The frequent over-expression of anti-apoptotic proteins such as Bcl-2 in BCCs (Cerroni and Kerl, 1994; Verhaegh *et al*, 1995; Crowson *et al*, 1996; Wikonkal *et al*, 1997; Chang *et al*, 1998; Miralles *et al*, 1998) may account for the lack of observed apoptosis. In summary, our findings reveal an inverse correlation between the presence of HPV DNA and apoptosis in SCCs which suggests that inhibition of apoptosis by HPVs may contribute towards the development of a proportion of these lesions.
